# Predicting Species’ Vulnerability in a Massively Perturbed System: The Fishes of Lake Turkana, Kenya

**DOI:** 10.1371/journal.pone.0127027

**Published:** 2015-05-19

**Authors:** Natasha J. Gownaris, Ellen K. Pikitch, William O. Ojwang, Robert Michener, Les Kaufman

**Affiliations:** 1 Institute for Ocean Conservation Science, School of Marine and Atmospheric Sciences, Stony Brook University, Stony Brook, New York, United States of America; 2 Kenya Marine and Fisheries Research Institute, Kisumu, Kenya; 3 Department of Biology, Boston University, Boston, Massachusetts, United States of America; 4 Conservation International, Arlington, Virginia, United States of America; University of Shiga Prefecture, JAPAN

## Abstract

**Background and Trophic Diversity Study:**

Lake Turkana is an understudied desert lake shared by Kenya and Ethiopia. This system is at the precipice of large-scale changes in ecological function due to climate change and economic development along its major inflowing river, the Omo River. To anticipate response by the fish community to these changes, we quantified trophic diversity for seven ecological disparate species (*Alestes baremose*, *Hydrocynus forskalli*, *Labeo horie*, *Lates niloticus*, *Oreochromis niloticus*, *Synodontis schall*, and *Tilapia zillii*) using stable isotopes. Based on their marked morphological differentiation, we postulated that dietary niches of these species would be similar in size but show little overlap. The degree of trophic diversity varied greatly among the species studied, refuting our hypothesis regarding dietary niche size. *Oreochromis niloticus* and *L*. *niloticus* had the highest trophic diversity and significantly larger dietary niches than *T*. *zillii*, *A*. *baremose* and *H*. *forskalli*. Low overlap among the dietary niches of the seven species, with the exception of the synodontid catfish *S*. *schall*, is consistent with our second hypothesis.

**Predicting Species’ Vulnerability:**

Breeding vulnerability was highest among those species with the lowest trophic diversity. We predict that in suffering two strikes against them, *A*. *baremose*, *H*. *forskalli*, *T*. *zillii*, and *L*. *horie *will be most affected by the highly altered Lake Turkana ecosystem and that *O*. *niloticus*, *L*. *niloticus* and *S*. *schall* will be least affected. Low vulnerability among *O*. *niloticus* and *L*. *niloticus* is promising for the future of the lake’s fishery, but the third most important fishery species (*L*. *horie*) will be highly vulnerable to impending ecosystem change. *T*. *zillii* should be treated as separate from *O*. *niloticus* in the fishery given higher sensitivity and a different ecological role. We see potential for expansion of the fishery for *S*. *schall *but don’t recommend the development of a fishery for *A*. *baremose* and *H*. *forskalli*.

## Introduction

Lake Turkana is an understudied rift valley lake located in northwestern Kenya that straddles the Ethiopian border. With a surface area of about 6,750 km^2^, it is Africa’s fourth largest lake and the world’s largest permanent desert lake [1]. Numerous tribes depend increasingly upon the lake’s fishery due to the unsustainable nature of their traditional livelihood of pastoralism in this arid region [2–3]. Lake Turkana is also a haven for wildlife, supporting over 350 native and migratory bird species and the world’s largest remaining population of the Nile crocodile [4]. Owing to the faunal diversity and paleoanthropological importance of the region, also known as the “cradle of mankind” [5], it has been named as a UNESCO World Heritage Site.

The Lake Turkana ecosystem is currently at the precipice of large-scale changes in ecological function due to multiple economic activities. These include building dams for hydroelectric power generation (the Gibe Dams) and large irrigation schemes along the Omo River, which supplies 90% of the lake’s water and has been called the lake’s “umbilical cord” [6]. The Gibe III Dam, the resevoir of which began filling in February, 2015, will be the largest hydropower project in Africa and the fourth largest in the world. In addition to lowering Lake Turkana’s water levels during reservoir filling, the dam will drastically reduce the magnitude of the lake’s flood cycle and therefore will likely impact the timing and success of fish breeding and migration [7–8]. In addition to the Gibe Dams, over 6,400 hectares of land were cleared for sugarcane and cotton plantations in the lower Omo Valley as of April 2014 [9]. Diversion dams, roads, survey lines, and other irrigation infrastructure are also being constructed [9]. When operational, these plantations will cover over 200,000 hectares. The associated large-scale irrigation schemes will consume substantial portions of the Omo River’s flow and could lead to lake level declines on the order of 20 meters in a lake only 30 meters deep on average [8]. Flow reduction can impact fish communities by eliminating spawning and nursery areas in the watershed and by altering food web dynamics through changes in species composition and basic limnological function. Due to these impending threats, Lake Turkana is currently under consideration as a World Heritage Site in Danger by the United Nations Environmental Program.

Changing climate conditions have not been considered throughout the planning of the hydropower and irrigation projects discussed above, but will influence their degree of impact on the Lake Turkana ecosystem [10]. In general, climate projections for Ethiopia suggest higher temperatures in the late 21^st^ century as compared to the late 20^th^ century and lower levels of precipitation [11]. Precipitation in the Omo Gibe basin specifically is predicted to decrease during the long rainy season and increase during the short rainy season and dry season [12–13].

The Lake Turkana fish community will be subjected to two phases of profound impact due to upstream development and climate change. Initially, as littoral habits shrink and flood pulse breeding cues diminish, species dependent upon these attributes will decline in abundance, with likely knock-on impacts to the lake food web as a whole. The overall productivity of the system will also change during this stage, as Lake Turkana is a nitrogen-limited “allothropic riverine lake”, meaning that it is heavily dependent on nutrient inputs from the Omo River [14–18]. In the second wave of impacts, species resilient to the initial changes will face increasing salinity and alkalinity as the lake’s volume declines further. These physiochemical changes will alter species dominance at multiple trophic levels [19–20]. Here we project likely impacts of changes in water inflow on seven key species in the Lake Turkana fish community (*Alestes baremose*, *Hydrocynus forskalli*, *Labeo horie*, *Lates niloticus*, *Oreochromis niloticus*, *Synodontis schall*, and *Tilapia zillii*) based on their dietary niche and breeding vulnerability.

We chose the species in this study based on the following criteria: 1. dominant species in the ecosystem, 2. species with economic and/or ecological importance, and 3. species representing different trophic guilds based on morphological differences (Table 1). Economic importance was gauged by the contribution of each species to fisheries catch using species composition data from previous studies spanning several years: 1960–1988 [21] (some years excluded due to a high percentage of unspecified catches), 2004 [22], 2005 [23], 2011 (KMFRI, pers. comm.). Records of fisheries catch were more consistent during the 1960’s-1980’s because of the Turkana Fishermen’s Cooperative Society, which collapsed in 1989 [22]. Ecological importance (top predators, trophic links, etc.) was gleaned from studies on the ecosystem conducted in the 1970’s and 1980’s [14,22] and on the role of these species in other ecosystems.

**Table 1:** Diet and Fishery Contribution of the Seven Species Studied.

Species	N	1960–2011 Average Portion of Catch (%)	2011 Portion of Catch (%)	Assumed Main Diet Component
*Alestes baremose*	73	0.5	3	Zooplankton
*Hydrocynus forskalli*	113	2.05	2	Fish
*Labeo horie*	77	16.48	14	Epibenthic algae/Detritus
*Lates niloticus*	111	16.45	16	Fish/Prawns
*Oreochromis niloticus*	114	27.89	43	Phytoplankton
*Synodontis schall*	92	2.46	3	Zooplankton/Insects/Benthos
*Tilapia zillii*	55	N/A	N/A	Macrophytes/Epilithic algae

On average, the species studied constituted 65.8% of the total fisheries catch from 1960–2011 (selected years) and 81% of the total fisheries catch in 2011. The catch listed for *O*. *niloticus* is that of all tilapia species in the lake combined (*O*. *niloticus*, *T*. *zillii*, and *Sarotherodon galilaeus*). Based on the species composition of heavily fished areas such as Ferguson’s Gulf, *O*. *niloticus* is likely to make up the majority of this catch [14]. The overall increase in the contribution of the species we studied to the fishery is primarily due to declines in the catch of *Citharinus citharus* and *Distochodus niloticus* over the 1960–2011 time period. *C*. *citharus* and *D*. *niloticus* were important fishery species in the 1970’s but their populations collapsed by the 1980’s [24].

Two of the most important fishery species by volume, *L*. *niloticus* and *O*. *niloticus*, are also the most valuable. The swim bladder of *L*. *niloticus* is a particularly coveted item and, at 166 ksh/kg, is worth more than five times that of bulk *L*. *niloticus* and *O*. *niloticus* [18]. Tilapia are targeted in shallow areas using seine nets and are a particularly important fishery resource during high production “boom” years. For example, Ferguson’s Gulf, with an area of only 10km^2^, yielded 16,000 tons of tilapia at its peak in 1976 [22].

Although not all of the study species made up a large portion of catch (e.g. *A*. *baremose*, *S*. *schall*), all play important ecological roles in Lake Turkana and, based on their morphological adapatations and previous research, are trophically disparate from each other. *Oreochromis niloticus* and *T*. *zillii* both possess deep bodies that accommodate long guts associated with an herbivorous diet, whilst *T*. *zillii* possesses tricuspid teeth unique among the tilapias, which allows it to consume macrophytic vegetation and to scrape epilithic algae [14,25–28]. Tilapiines are the most widely distributed group of non-native fishes [29], including established populations of four exotic species in Lake Victoria. *Oreochromis niloticus* is native to Lake Turkana where it plays important ecological and economic roles. It is the only species in the lake that can both concentrate and digest the blue-green algae *Microcystis aeruginosa*, one of the lake’s dominant phytoplankton species. Thus it links and transfers the otherwise wasted (from a fisheries standpoint) energy (i.e. primary production) to higher trophic levels [14,16,30]. While not distinguished from *O*. *niloticus* in Lake Turkana’s fishery, *T*. *zillii* is likely to prove distinct in terms of its resilience to perturbation due to its trophic dependence on macrophytic vegetation.

*Alestes Baremose*, along with other species of *Alestes* and *Brycinus* spp. is thought to act as an important conduit of energy from zooplankton to piscivorous fishes in Lake Turkana [14]. This species exhibits the hallmark body and caudal form of a midwater swimmer, and the small mouth of a zooplanktivore [14]. *Synodontis schall* and *L*. *horie* have morphological characteristics consistent with a benthivorous diet, including barbels and a sub-terminal mouth. While *S*. *schall*’s buccal jaw possesses teeth that are sometimes employed to crush the shells of benthic invertebrates, like all cypriniforms, *L*. *horie* lacks buccal dentition (there are dentigerous pharyngeal jawplates) exhibiting instead a highly folded buccophayngeal membrane that facilitates the efficient entrapment of detrital particles [14,31]. The pelagic food web of Lake Turkana is largely detritus-based and the lake is home to several detritivorous fish species [1]. *L*. *horie* was chosen to represent the detritivore fish group due to its importance to the lake’s fishery. As an ecological generalist [32–35], *S*. *schall* may play an important role in Lake Turkana by expanding into portions of the food web vacated by the decline of other species. Lake Turkana harbors a unique midwater scattering layer of endemic characoids (*Brycinus* spp) that have shown population declines linked to reduced lake level [1,14,36]. It is possible that *S*. *schall* has expanded its distribution to assume some of their role in the food web, an idea supported by the dominance of this species in recent gill net surveys of the open waters, and by the persistence of this species during the population declines of *Brycinus* spp. in the 1970’s-1980’s [37]. *Synodontis schall* may also be a food web “dead end”, as it is not readily consumed by other species due to its elongate, locking dorsal and pectoral spines [1,14,37].

*L*. *niloticus* is a top predator in many African Lake ecosystems, including in Lake Victoria where it is an invasive species that contributed to the decimation of native cichlid populations [38–41]. *L*. *niloticus* has been found to impact diversity and food web length in the Lake Kyoga ecosystem and may also exert important top down control in Lake Turkana [41]. *H*. *forskalli*, a highly piscivorous apex predator in Lake Albert, the African Lake with the most similar fish assemblage to Lake Turkana, likely plays a similar role in this ecosystem [42].

*Lates niloticus* and *H*. *forskalli* exhibit different morphological attributes indicative of a predatory lifestyle. In the case of *L*. *niloticus*, a large gape size allows for the consumption of prey items up to 50% of their body length [14] and the presence of a tapetum lucidum enhances hunting in low light conditions [43]. *Hydocynus forskalli*, while lacking a large gape, has interlocking razor-like teeth that allow it to tear prey items and an elongate body to allow for fast swimming to pursue prey [14,44].

There has been growing interest in using dietary position and breadth as measured by stable isotopes to explore trophic niche relationships and the ecological niche concept [45–54]. Stable isotope analysis confers benefits over gut content analysis because it provides a measure of what is digested rather than ingested and integrates diet over time periods ranging from days to years, depending on the metabolic activity of the tissue sampled [55–60]. Particularly useful are several quantitative measures developed and refined over the last several years that allow researchers to compare trophic diversity among populations and communities using stable isotope data [47,53–54,61–66].

We used stable isotopes to measure the degree of intraspecific diet variation, as populations of specialist species (small ecological niche) are expected to be more vulnerable to perturbation than populations of generalist species (large ecological niche) [67]. Disproportionate declines in specialist species has been documented for a suite of taxa worldwide, from fungi to mammals [68–69,70–76]. Fossil records show that specialist species are more likely to go extinct than are generalist species over geological time scales [77–78]. When specialist species decline, they are replaced by a relatively small number of generalist species, lowering biodiversity via “taxonomic homogenization” [79–81]. Although the ecological niche by definition is “n-dimensional”, the isotopic niche width, a measure of dietary niche breadth, is a useful proxy for understanding ecological niche where δ^13^C and δ^15^N represent environmental and trophic axes, respectively [50,53,82]. Other factors can influence isotopic variance [83], but a large isotopic niche may indicate a generalist population of specialists (Type B generalism; [46]) and variation in isotopic signature is correlated with intraspecific trophic diversity as measured by gut content analysis [84].

To consider how water inflow changes will influence the breeding success of the species studied, we developed a breeding vulnerability index that combines species-specific information on flood pulse dependence and breeding habitat [14]. In tropical and neotropical freshwater systems, it is a common phenomenon for fishes to show breeding peaks that coincide with periods of spate [7,85–88]. Given likely changes in the flooding regime of Lake Turkana, the degree to which its fishes depend on the flood pulse as a breeding cue will influence their vulnerability. As lake level declines, habitat availability will also be altered, with the most immediate declines occurring on the lake’s rocky eastern shores, which harbor extensive macrophyte beds, and in the Omo Delta region [10]. Breeding habitat preference is therefore another important component of breeding vulnerability.

Based on strong evolutionary specialization and differences in gut contents among the species studied, we postulate that their dietary niches: 1. will not be significantly different in size and 2. will occupy different places in isotopic space and therefore show low overlap. In testing these hypotheses, our goal is to develop a better understanding of the relative dietary niche breadths of these seven key species. From these data and an understanding of the breeding vulnerability of the species studied, some inferences can be made regarding the effects of greatly reduced flow from the Omo River, with implications for the lake’s fishery. Although the ecological implications of intraspecific diet variation for populations, particularly in terms of fitness and adaptability, have been noted elsewhere [89], this is the first study to use intraspecific diet variation as a predictor of response to perturbation, as well as the first study to apply these measures to the Lake Turkana ecosystem. Our results serve as a robust initial prediction of how Lake Turkana’s fish community will be altered by upstream development.

## Materials and Methods

All field sample collection was conducted with minimal discomfort to the animals of study. Our methodology was reviewed and approved by Stony Brook’s Institutional Animal Care and Use Committee (Project 262729). We also obtained a permit (NCST/RRI/12/1/BS011/99) to conduct our field work in Kenya, through the National Council for Science and Technology (now known as the National Commission of Science, Technology and Innovation-NACOSTI), and collaborated with several local organizations, including the Kenya Marine and Fisheries Research Institute, to minimize any potential negative impacts of the research.

We collected fish samples from Lake Turkana, Kenya using gill nets at various sites between 2008 and 2012. With each net set, we sampled 10 individuals of every species, ensuring to capture a representative size range. A small piece of epaxial muscle tissue was removed from each individual and placed in the sun until fully dry (average air temperature of 31–33°C, arid conditions), then stored in a cryovial until processing.

**Fig 1:**
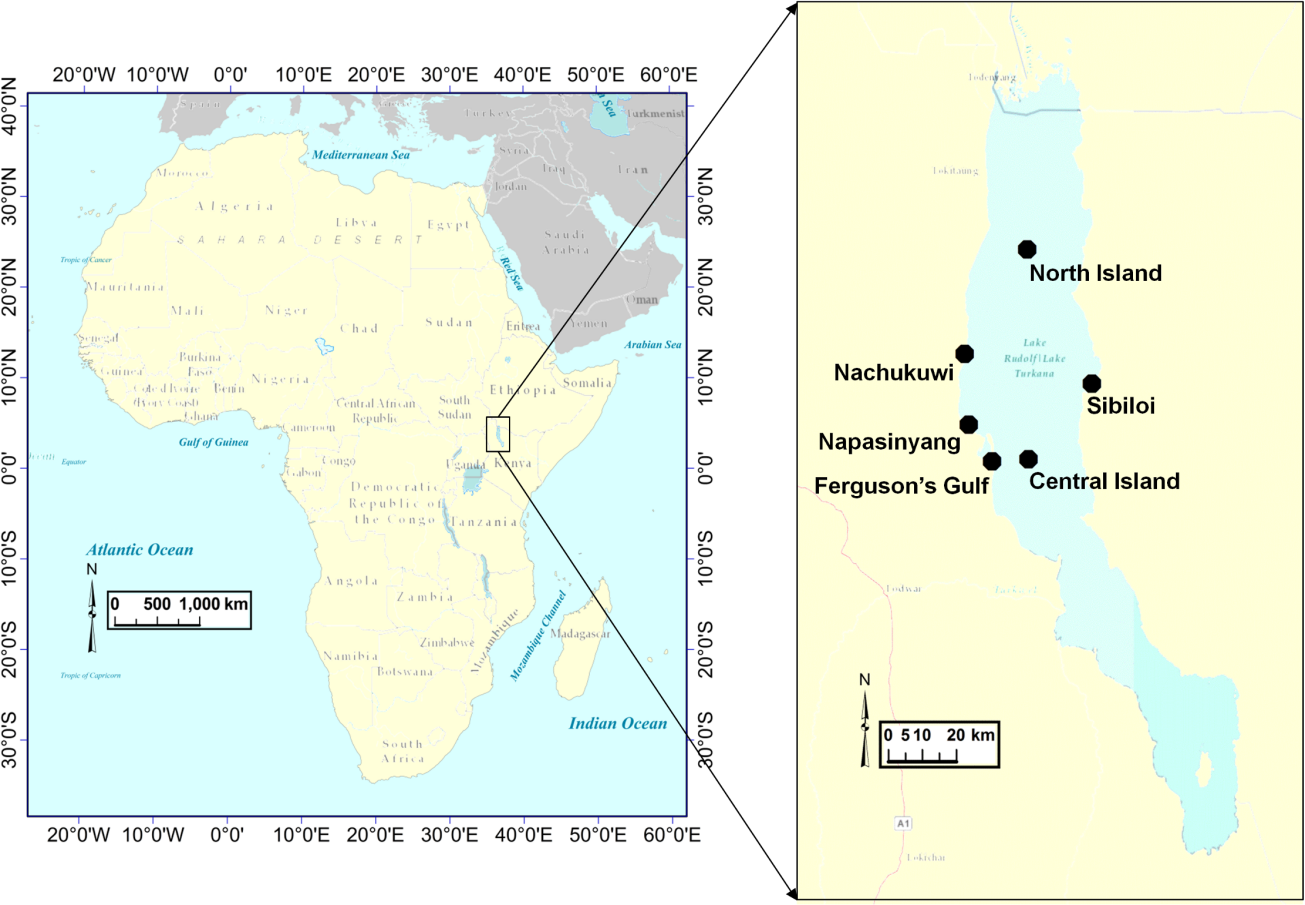
Map of Africa with an inset of Lake Turkana showing the study’s six sampling sites.

Sample sites varied from year to year due to inter-tribal conflict in some areas during later years. In 2008, we sampled in May and December, respectively. In May 2008, we sampled three sites: open waters near Northern Island at depths of approximately 30m, open waters near Central Island at depths of approximately 30m, and a littoral site, Nachukui. In December 2008, we sampled two bays in Sibiloi National Park, located on the eastern shores of the lake. Shallow areas within the confines of Sibiloi National Park are protected from fishing and harbor the largest macrophyte beds (dominated by *Potamogeton* spp.) in the lake proper, which function as an important nursery habitat [90]. We were unable to collect samples again until March and December of 2011. In March 2011, we sampled Napasinyang, an ephemeral river mouth on the western shore of the lake. In December 2011, we sampled two bays in Sibiloi National Park and at the mouth of Ferguson’s Gulf. Ferguson’s Gulf is the lake’s most productive fisheries area and an important breeding habitat, particularly for *O*. *niloticus*, and our sampling suggests that predatory fishes (including *H*. *forskalli* and *L*. *niloticus*) frequently move into the gulf to feed on juvenile fishes. The final sampling trip took place in July of 2012, when we sampled three areas of Ferguson’s Gulf (the mouth of the Gulf, a grassy area of the Gulf and a mid-section of the Gulf), two bays in Sibiloi, and open waters near Central Island. The grassy area of Ferguson’s Gulf is dominated by hippograss, *Echinochloa stagnina*_,_ rather than by submerged macrophytes. Our sampling sites were chosen to represent different habitat types in Lake Turkana, including latitudinal differences in habitat (e.g. Nachukui to Central Island), heavily fished (e.g. Ferguson’s Gulf) versus unfished (e.g. Sibiloi) habitats, littoral (<5m; Napasinyang, Ferguson’s Gulf, Sibiloi, and Nachukui) and pelagic habitats (Central Island and Northern Island), phytoplankton (e.g. Ferguson’s Gulf) and macrophyte (e.g. Sibiloi) dominated habitats, and a spectrum of low conductivity (e.g. North Island) to high conductivity (e.g. Ferguson’s Gulf) habitats (Fig 1).

We homogenized samples using a mortar and pestle cleaned with 70% ethanol. Samples were then weighed to the nearest thousandth of a milligram and analyzed for C:N, δ^15^N, and δ^13^C using a GV Instruments IsoPrime isotope ratio mass spectrometer. We calibrated all values to the international standards Vienna Pee Dee belemnite for carbon and atmospheric air for nitrogen. To determine intra-sample variability, one duplicate sample was run per every 10 unique samples. An internal laboratory standard sample was also run alternating between glycine and peptone for every 10 unique samples to test for machine accuracy. Both standards have been carefully calibrated using traditional methods (Dumas combustion, dual inlet IRMS) and have been calibrated to IAEA standards N1 and N2 for nitrogen, and NBS 20, 21 and 22 for carbon. The average observed values of glycine were -33.95±0.06 for δ^13^C and 10.77±0.13 for δ^15^N (n = 36) and agreed well with the expected values for this standard of -34.00 for δ^13^C and 10.73 for δ^15^N (average absolute difference of 0.07± 0.05‰ for δ^13^C and of 0.10±0.10‰ for δ^15^N). The average observed values of peptone were -14.75±0.07 for δ^13^C and 7.28±0.13 for δ^15^N (n = 39) and agreed well with the expected values for this standard of -14.73 for δ^13^C and 7.40 for δ^15^N (average absolute difference of 0.06± 0.05‰ for δ^13^C and of 0.15±0.10‰ for δ^15^N). A total of 57 pairs of duplicates for the species studied were analyzed and showed good agreement for δ^13^C (average absolute difference of 0.06±0.06) and δ^15^N (average absolute difference of 0.24±0.19).

Lipid extraction is a necessity in some SIA studies [91–93] because lipids tend to show depleted and variable δ^13^C values as compared to the rest of the organism [94–95]. However, the process of removing lipids can also impact the δ^15^N value of a sample [94,96] To circumnavigate this issue, several mathematical lipid correction models have been created that rely on the relationship between C:N and the change in δ^13^C due to lipid removal [95,97–99]. In general, aquatic stable isotope samples with C:N<3.5 have low lipid concentrations (<5%) and are not altered much by lipid extraction [98]. Our samples had an average C:N of 3.05±0.23, with only six samples exceeding a C:N of 3.5. To be certain that we could use untreated δ^13^C values that did not require lipid extraction or the use of lipid correction models, we conducted a preliminary lipid extraction study.

We randomly chose a subset of 28 samples to analyze pre- and post-lipid extraction, using a variation of the methods described in [100] and [101]. For each sub-sample, we placed 2–5 mg (depending on sample size) into a cryovial, which we then filled with a 2:1 chloroform:methanol solution. We vortexed the samples then placed them in a 30°C water bath for 24 hours. On the second day of the procedure, we centrifuged the cryovials for 5 minutes and decanted the supernatant. We repeated the procedure for each sample to remove any remaining lipids. We dried the samples under a fume hood for 48 hours before processing them for SIA. For comparison purposes, we also analyzed a sub-sample pre-lipid extraction (bulk sub-sample) that corresponded with each lipid extracted sub-sample. To determine whether lipid extraction would improve the quality of our results, we tested whether ∆δ^13^C for the 28 samples correlated with their C:N ratio. The ∆δ^13^C was defined as $\Delta \delta^{13}C=\left(\frac{\left(\delta^{13}C_{B}-\delta^{13}C_{LE}\right)}{\delta^{13}C_{B}}\right)*100$, or the percent change in the sample’s isotope signature, where δ^13^C_B_ represents the isotope signature of the bulk sub-sample and δ^13^C_LE_ represents the isotope signature of the lipid extracted sub-sample. If ∆δ^13^C resulted from the loss of lipids alone, a positive correlation would be expected because lipids do not contain nitrogen, so C:N is correlated with the lipid content of a tissue. We also examined ∆δ^15^N, defined in the same manner as ∆δ^13^C, to determine if this isotope’s signature was impacted by the lipid extraction procedure.

Neither ∆δ^13^C (r^2^ = 0.02, p<0.54) nor ∆δ^15^N (r^2^ = 0.01, p<0.60) showed a significant positive correlation with C:N, but the changes in the two isotopes were positively correlated with each other (r^2^ = 0.68, p<1.2x10^-07^). Furthermore, for most of our samples the absolute value of ∆δ^15^N (average of 15.42±19.11%) was greater than the absolute value of ∆δ^13^C (5.13±5.39%) and both isotopes showed large standard deviations. Due to a low C:N among the samples, the lack of a consistent relationship between ∆δ^13^C and C:N, and the impact of lipid extraction on ∆δ^15^N, uncorrected δ^13^C signatures were used for the remainder of the study.

### Trophic Diversity and Overlap

To answer questions regarding isotopic niche, the standard ellipse function in the Stable Isotope Analysis in R (SIAR) package, known as Stable Isotope Bayesian Ellipses in R (SIBER) was used [53,102]. The standard ellipse area, SEA, utilizes the δ^15^N /δ^13^C covariance matrix and is akin to standard deviation for univariate data [53]. This ellipse includes approximately 40% of the data cloud and can be taken to represent a core isotopic niche. To reduce the influence of small sample sizes, a correction is made to the SEA value (i.e. using a correction of n-2 rather than n-1 for estimates of variance and covariance due to the use of two-dimensional data), resulting in SEA_c_ [53]. Hereafter, we will refer to SEA_c_ as the isotopic niche. A Monte-Carlo stimulation build into SIAR is used to produce a range of possible values for the isotopic niche area of each species and to account for uncertainty in the data. We refer to the mean from this stimulation as SEA_B_. We used these Bayesian estimates to calculate the probability that one species’ isotopic niche was greater than another species’. Using SIAR, we also estimated the percent overlap between two isotopic niches.

The SIAR package can also be used to calculate Layman’s Metrics [47]. Layman’s Metrics were developed to compare communities or populations in terms of trophic diversity and include estimates of isotopic niche (Convex Hull- Hull) and measures of dispersion in isotope space (Centroid Distance- CD; Mean Nearest Neighbor Distance- MNND; Standard Deviation of Nearest Neighbor Distance- SDNND). Due to the sample size dependent nature of these metrics [40–41], we bootstrapped (R = 1005) them for the minimum sample size among the species studied (n = 50; minimum sample size was for *T*. *zillii*, where n = 55). For each metric, we recorded the average from the bootstrapping exercise, indicated by the subscript “b”.

### Baseline Signature Variability

To better understand the variability in baseline isotope signatures among sites, we collected plankton samples in four size fractions (<20μm, 20–90μm, 90–250μm, >250μm) at all sites in 2011 and 2012 using nylon filters. We rinsed this material onto pre-combusted GFF filters using deionized water and dried the filters in the sun. To compare baseline isotope signatures across size classes and sites, we ran ANOVA and Tukey’s Post-Hoc test. These tests, in addition to all subsequent statistical tests discussed, were conducted in R version 2.15.1 [102]. We collected a water sample in association with each plankton sample and preserved it using a 2% Bouin’s solution. Upon return to Stony Brook University, we gently mixed each sample and analyzed 0.1 ml for community composition using a FlowCAM.

Phytoplankton are often inadequate baseline organisms, given their large spatial and temporal variability in isotopic signature [103–105]. Filter feeders, grazers and other primary consumers can act as effective baseline organisms because they integrate primary producer signatures over longer time periods and larger spatial extents [92]. Lake Turkana, however, has a “depauparate” invertebrate fauna and therefore lacks ubiquitous and appropriate baseline primary consumers [106]. An alternative approach is to supplement baseline signatures derived from phytoplankton with baseline signatures derived from attached primary producers (e.g. macrophytes). Though attached primary producers are found in some areas of Lake Turkana, they vary from site to site and are not found at all sites. Of the sites sampled, hippograss (*E*. *stagnina*) dominates in Ferguson’s Gulf but can also be found in Sibiloi, the perennial herb *Typha domingensis* is found only at Napasinyang, and the macrophyte *Potamogeton* spp. is found only at Sibiloi.

We addressed the issue of suboptimal baseline signatures and their influence on variability at higher trophic levels in several ways. First, we collected samples of attached vegetation despite its limited application across sites and tested for significant differences where appropriate. Second, we conducted several analyses to better understand how baseline signatures may have impacted the size of the isotopic niches of the species studied. We ran size-corrected linear models (i.e. on the residuals resulting from size-signature regressions) on each species to determine what proportion of the remaining variability in their isotope signature was related to site, year, and their interaction terms. The use of size-corrected models allowed us to explore the influence of baseline variability without the confounding factor of differences in the size range of a species across sites and years (e.g. *O*. *niloticus* sampled in Ferguson’s Gulf were much smaller on average than those sampled in Sibiloi). If baseline differences were large and influential, we would expect the variability described by these factors to be high and relatively consistent across species. Conversely, if the amount of variability described by these factors was inconsistent across species, this variability is more likely the result of differences in prey items consumed across space and time. We also explored the relationship between intraspecific δ^13^C variability and δ^15^N variability, respectively, described by these factors. If variability attributed to site, year and their interaction terms resulted primarily from baseline differences, we would expect a positive relationship between the variability described for δ^13^C and δ^15^N. Lastly, we calculated the number of unique sites at which each species was sampled and explored the relationship between this number and the isotopic niche size of each species. We defined unique sites as any combination of site and year for which >5 (10% of the smallest sample size) individuals of a species were sampled. If spatial or temporal baseline differences played a major role in determining the overall variability of a species’ isotopic signatures and therefore the size of their isotopic niche, we would expect a positive relationship between the number of unique sites and isotopic niche size.

### Breeding Vulnerability Index

To consider our intraspecific trophic diversity results in the context of breeding vulnerability, we developed an index based on the breeding behaviors of Lake Turkana’s fishes [14]. This index is a summation of the scores for two factors, flood pulse dependence and breeding habitat (Table 2). Under the habitat factor, species breeding in pelagic habitats are expected to be least impacted by changes in water inflow, while those breeding in the Omo River or Omo Delta exclusively will be the most strongly impacted. Among the species breeding in shallow areas, those that breed on the sandy, gently sloped western shores of the lake will be less impacted by water level declines than those that breed on the rocky, steep sloped eastern shores where new shallow habitats are less likely to emerge. Species capable of spawning in ephemeral rivers, including the Kerio and Turkwell Rivers, should not be as highly impacted as those spawning solely in the Omo River. Under the flood pulse factor, species that only breed during periods of spate (annual flood period of June-October) will be most vulnerable to changes in the Omo River’s flood regime, species that show strong breeding peaks during periods of spate will be moderately vulnerable, and species that show weak breeding peaks during periods of spate or consistent breeding year-round will be least vulnerable (Table 2). Partial scores were sometimes assigned within factors (e.g. if a species has two sub-populations that each breed in a different habitat). The overall breeding vulnerability index score ranges from 0 (low vulnerability) to 4 (high vulnerability).

**Table 2:** Breeding Vulnerability Index Factor Categories and Their Scores.

	Categories	Description	Score
Flood Pulse Dependence			
	Critical	Breeds exclusively during periods of spate.	2
	Moderate	Ripe females ≥50% more abundant during periods of spate.	1
	Low	Consistent breeding year-round or ripe females ≤50% more abundant during periods of spate.	0
Breeding Habitat			
	Most Threatened	Eastern Shore Shallow Areas (steep bathymetry) or Omo River	2
	Threatened	Western Shore Shallow Areas (gradual bathymetry) or All Rivers	1
	Least Threatened	Pelagic	0

## Results

### Trophic Diversity and Overlap

Among the fish species studied, δ^13^C ranged from -26.35 (*L*. *niloticus*) to -13.59 (*T*. *zillii*) and δ^15^N ranged from 1.53 (*O*. *niloticus*) to 16.71 (*H*. *forskalli*). The means of δ^13^C and δ^15^N were largely consistent with the end members for the ranges of these signatures, with *A*. *baremose* exhibiting the lowest average δ^13^C and *T*. *zillii* the highest. *O*. *niloticus* showed the lowest average δ^15^N and *H*. *forskalli* the highest (Table 3).

**Table 3:** Mean ± SD of Isotopic Signatures, Isotopic Niche and Layman’s Metrics for the Seven Species Studied.

Species	N	Mean δ^13^C	SD δ^13^C	Mean δ^15^N	SD δ^15^N	SEA_c_	Hull_b_	CD_b_	MNND_b_	sdMNND_b_
*Alestes baremose*	73	-20.18	1.31	11.34	1.93	6.47	25.56	1.98	0.30	0.23
*Hydrocynus forskalli*	113	-19.35	1.07	13.26	2.54	6.35	55.11	2.06	0.29	0.57
*Labeo horie*	77	-18.82	1.42	6.66	2.18	9.53	37.20	2.26	0.30	0.21
*Lates niloticus*	111	-18.46	2.86	10.93	2.86	12.58	62.00	2.99	0.33	0.72
*Oreochromis niloticus*	114	-17.21	1.69	5.03	2.61	10.37	56.80	2.72	0.31	0.38
*Synodontis schall*	92	-18.20	1.49	9.28	2.19	8.62	44.48	2.23	0.34	0.26
*Tilapia zillii*	55	-16.68	1.33	7.22	1.39	5.47	29.64	1.50	0.44	0.52

There was a significant positive correlation between the sample size of each species and both their niche volume (Convex Hull; r^2^ = 0.83, p<0.005) and centroid distances (r^2^ = 0.60, p<0.05). MNND had a significant negative correlation with sample size (r^2^ = 0.40, p<0.005), while there was no significant relationship between sdMNND and sample size. Once bootstrapped, the dependence of these metrics on sample size largely disappeared, with positive and borderline significant correlations for CD_b_ (r^2^ = 0.59, p = 0.045) and Hull_b_ (r^2^ = 0.57, p = 0.049). SEA_c_, showed no significant relationship with sample size (r^2^ = 0.33, p = 0.18).

**Fig 2:**
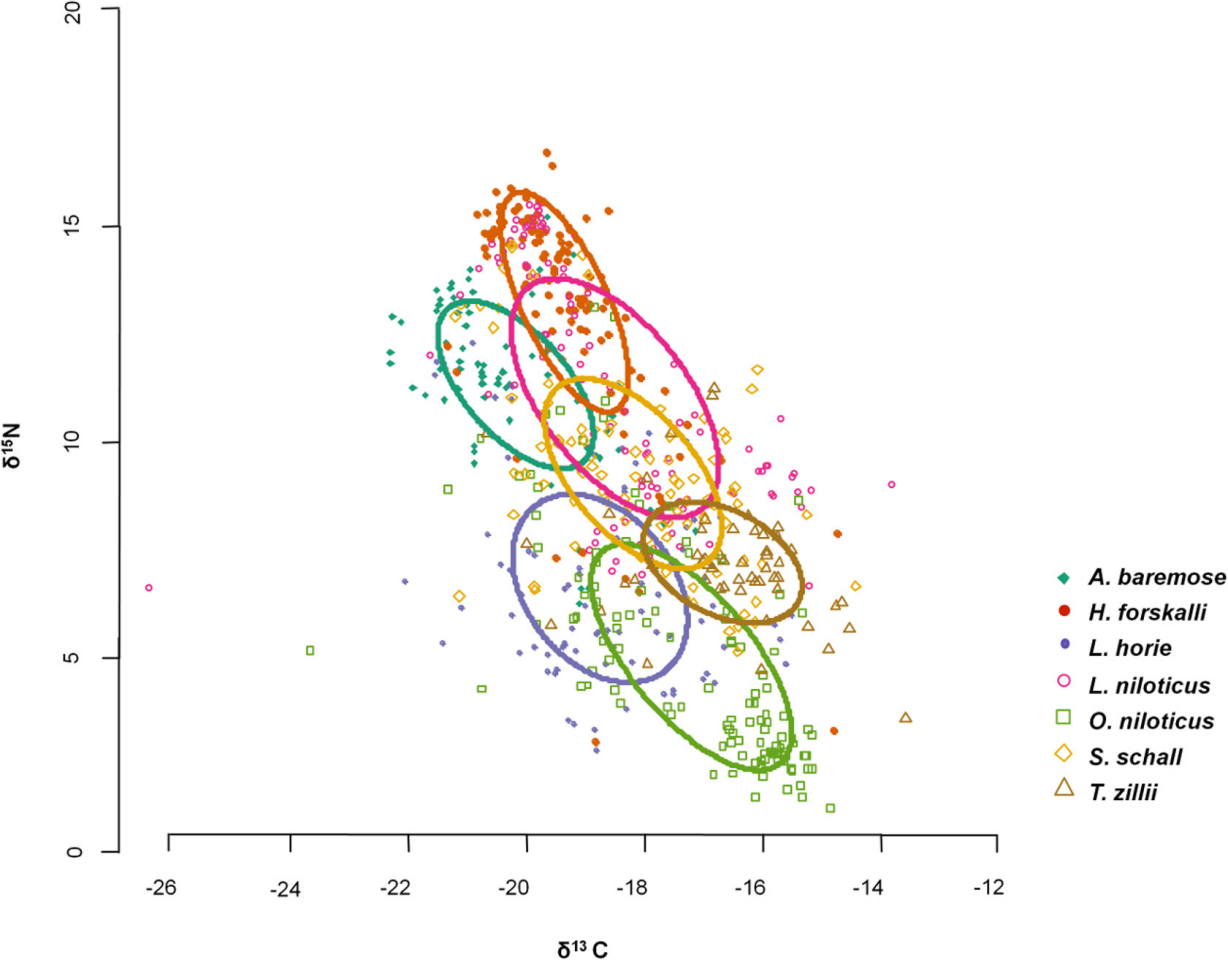
Isotopic niches for the seven fish species examined in this study. Symbols represent individual isotope values within species. Isotopic niches were calculated as standard ellipses in R, using the δ^13^C and δ^15^N signatures for each species.

**Fig 3:**
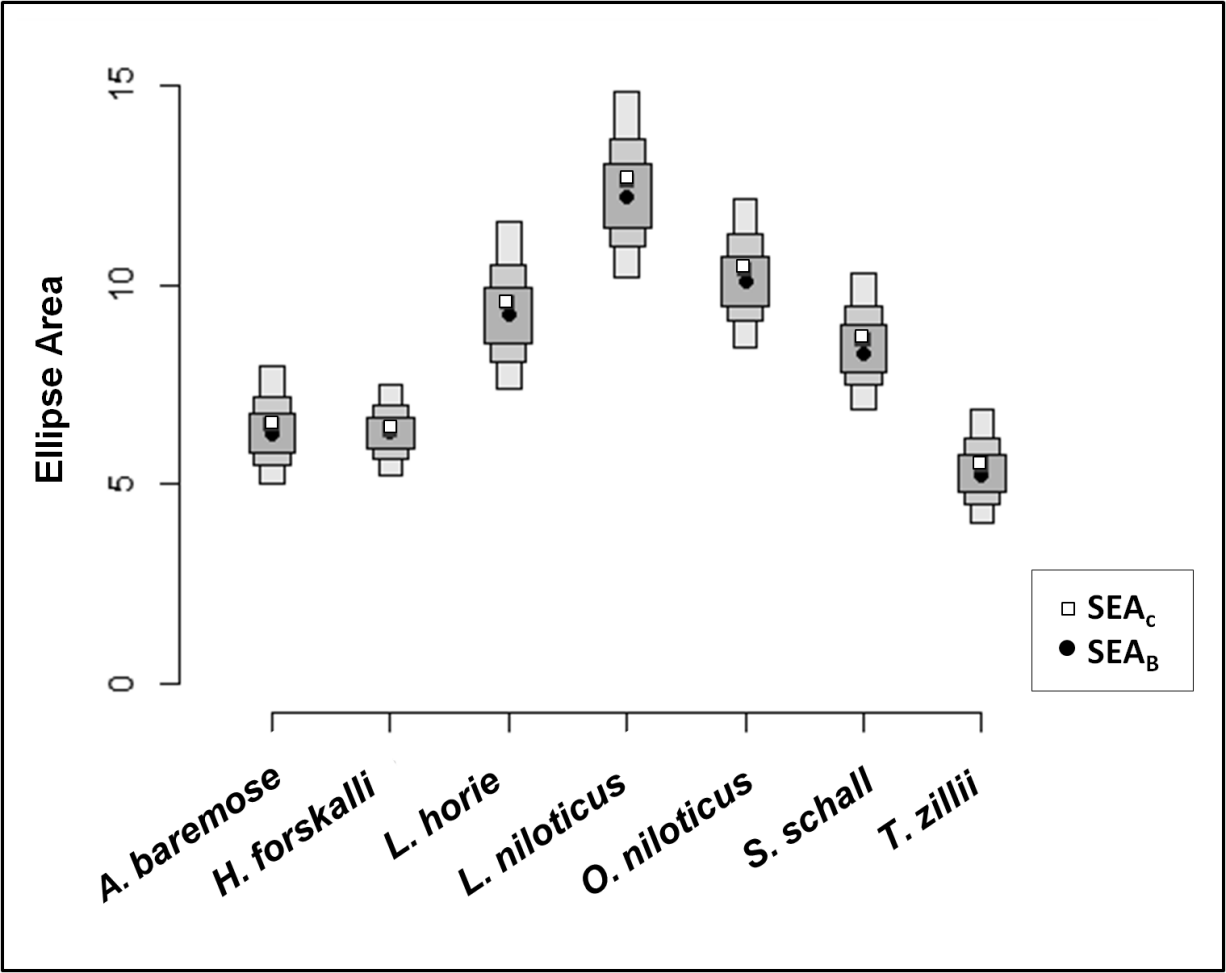
Box-plot of the Monte-Carlo stimulation for isotopic niches in R. This simulation accounts for the uncertainty in the isotope data and sizes of the isotopic niches. The black dot in each species’ box-plot represents the average isotopic niche size from the Monte-Carlo stimulation, SEA_B_, while the white box represents the SEA_c_ isotopic niche value.

SEA_c_ varied by a factor of two for the species studied and was smallest for characids (*H*. *forskalli* and *A*. *baremose*) and *T*. *zillii* and largest for *O*. *niloticus* and *L*. *niloticus* (Table 3, Fig 2). There was good agreement between SEA_c_ and SEA_B_ for each species studied (Fig 3). Confidence in the relative sizes of the isotopic niches was high (Table 4); i.e. when comparing species with the smallest isotopic niches (*A*. *baremose*, *H*. *forskalli*, and *T*. *zillii*) to those with the largest (*O*. *niloticus* and *L*. *niloticus*), probabilities were always >0.95 (Table 4). The results for bootstrapped Layman’s Metrics were in agreement with the results from SIBER (Table 3). In general, species with the largest SEA_B_ also had the largest Hull_b_ (with the exception of *H*. *forskalli*) and the highest values for the measures of dispersion CD_b_, MNND_b_, and sdMNND_b_

**Table 4:** Bayesian Probability for Isotopic Niche Size Comparisons.

	*Alestes baremose*	*Hydrocynus forskalli*	*Labeo horie*	*Lates niloticus*	*Oreochromis niloticus*	*Synodontis schall*	*Tilapia zillii*
*Alestes baremose*	x	0.48	0.99	1	1	0.96	0.17
*Hydrocynus forskalli*	0.52	x	1	1	1	0.98	0.15
*Labeo horie*	0.01	0	x	0.97	0.73	0.27	0
*Lates niloticus*	0	0	0.03	x	0.08	0	0
*Oreochromis niloticus*	0	0	0.27	0.92	x	0.09	0
*Synodontis schall*	0.04	0.02	0.73	1	0.91	x	0
*Tilapia zillii*	0.83	0.85	1	1	1	1	x

Rows- Probability that one isotope niche is smaller than another; Columns- Probability that one isotopic niche is larger than another.

The average eccentricity of the ellipses representing the isotopic niches was 0.87 ± 0.07, showing strong deviation from a perfectly circular shape. For all species, ellipse length was greater along the δ^15^N axis than along the δ^13^C axis. The only species with eccentricities below the average were *T*. *zillii* (0.75) and *L*. *horie* (0.81), suggesting that carbon source plays a more important role in the isotopic niche size of these species than the others studied. To account for the fact that larger variability along the δ^15^N axis could be due to a substantially larger average fractionation factor for this isotope relative to δ^13^C [79], we re-calculated the ellipses for each species using standardized values via the standard score method. The eccentricity trends were the same for the standardized ellipses, with an average of 0.88 ± 0.04 and with *L*. *horie* (0.79) and *T*. *zillii* (0.87) showing the smallest eccentricities.

The overlap between isotopic niches can be used as an indicator of functional redundancy or competition between species in an ecosystem [61,63]. We did not expect functional redundancy to be well-represented in this study, as the species examined were selected to represent the full suite of trophic guilds among fishes in Lake Turkana (Table 1). However, if a large portion of the isotopic niche of one species overlaps with multiple species from varying trophic guilds, omnivory is indicated.

The median percent overlap between any one species and another was low for *A*. *baremose* (1.15%), *H*. *forskalli* (1.33%), *L*. *horie* (2.10%), and *O*. *niloticus* (1.09%). Median percent overlap was slightly higher for *T*. *zillii* (6.23%) and highest for *L*. *niloticus* (10.63%) and *S*. *schall* (14.81%). Most species overlapped to some extent with three (*A*. *baremose*, *H*. *forskalli*, *L*. *horie*, and *O*. *niloticus*) or four (*T*. *zillii* and *L*. *niloticus*) of the other six species studied, whereas *S*. *schall* overlapped with all other six species. To further explore the overlap between *S*. *schall* and the other species studied, an aggregate isotopic niche was calculated (i.e. including all data from the species studied except for *S*. *schall*). The aggregate isotopic niche was large (SEA_c_ = 17.51), as might be expected due to the aggregation of several trophic guilds, and 97% of the isotopic niche of *S*. *schall* lay within this aggregate niche.

### Baseline Signature Variability

**Fig 4:**
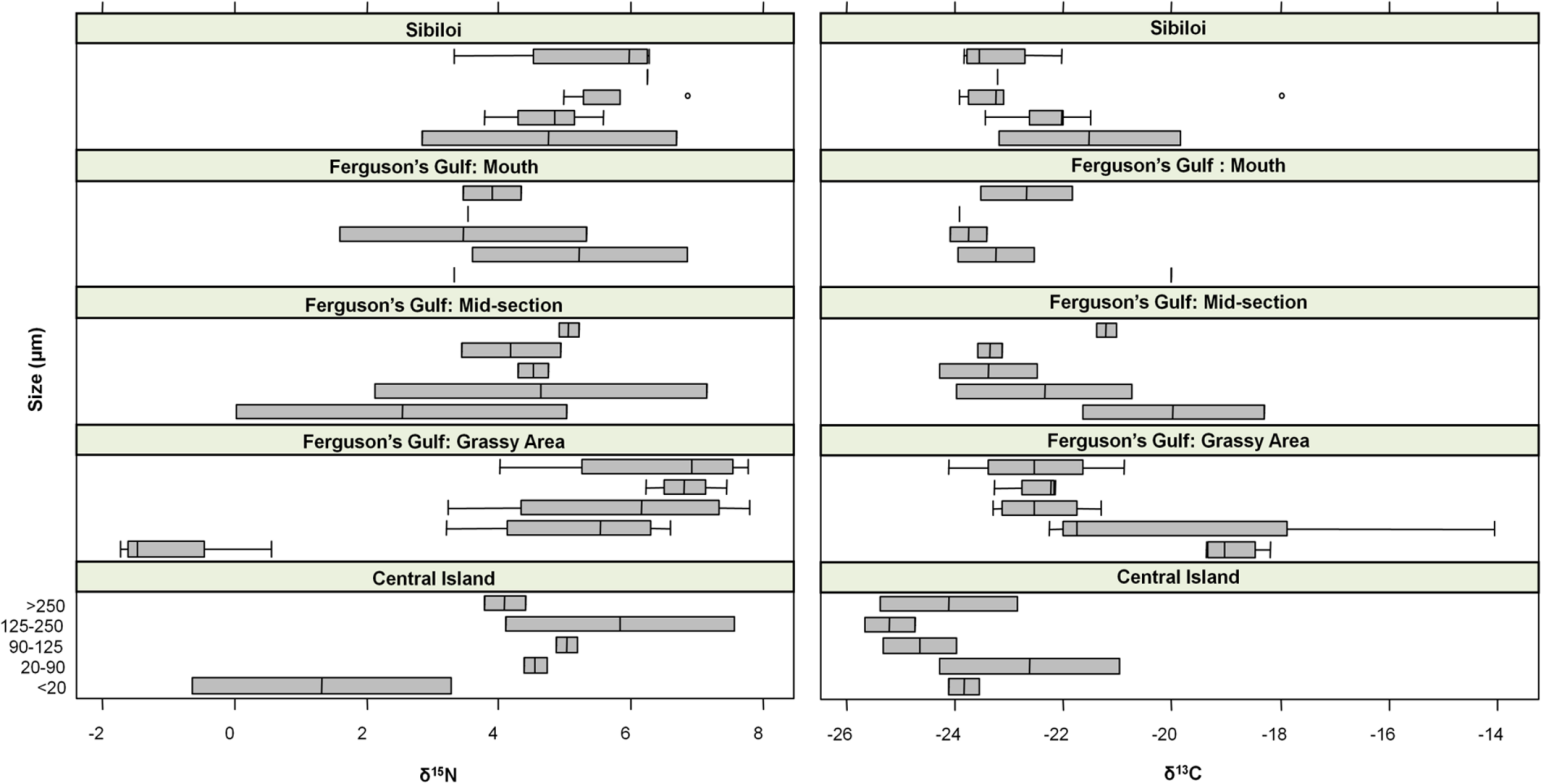
Boxplot of phytoplankton baseline isotope signatures at the study’s six sampling sites. Plankton samples were collected in five size fractions at each sampling site. Significant differences were found between the <20μm size class and all other size classes for δ^13^C and δ^15^N. Significant differences were found between the grassy gulf and open lake sites for δ^13^C.

Phytoplankton baseline signatures for δ^13^C (F = 6.88, P = 0.01) and δ^15^N (F = 12.81, P = 1.96x10^-07^) in the <20μm size class differed significantly from all other plankton size classes respectively (Tukey’s post-hoc test, Fig 4). All size classes were dominated by *M*. *aeruginosa* varying from single cells to large colonies, which would explain the similar isotopic signatures observed for all sizes classes >20μm. The presence of non-photosynthetic bacteria may explain the significantly different signature for the <20μm size class. Baseline signatures for δ^13^C differed among sites (F = 2.64, P = 0.02), but δ^15^N signatures did not (F = 0.417, P = 0.02). Tukey’s post-hoc test showed that the significant difference in δ^13^C was driven by the difference in signature between grassy areas of Ferguson’s Gulf and the open lake. There was no significant difference in hippograss baseline signatures between Sibiloi and Ferguson’s Gulf (S1 Fig).

**Fig 5:**
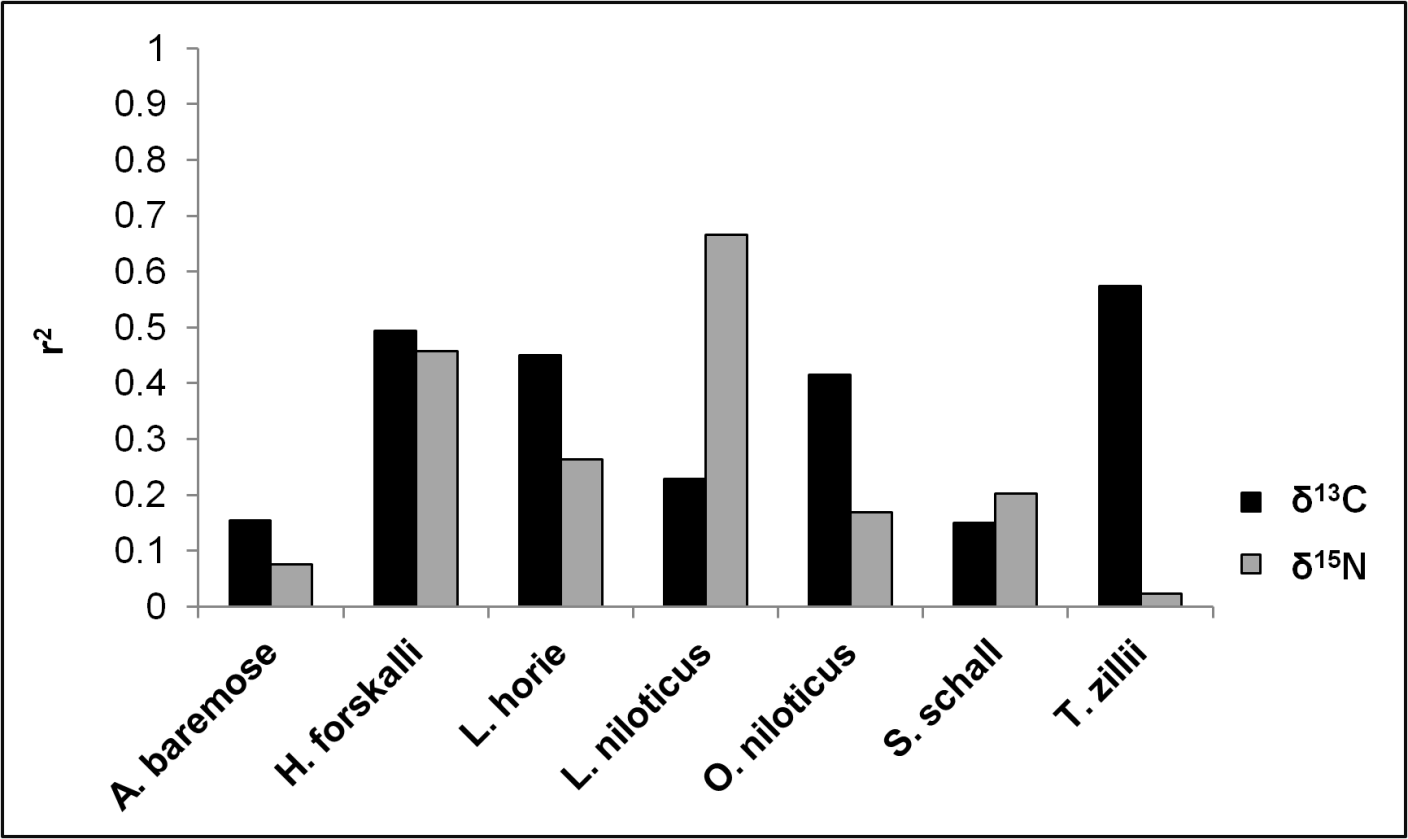
The amount of variation in isotopic signature (r^2^) described by site, year and their interaction terms. These values were calculated using size-corrected multivariate models, i.e. were run on the residuals of regressions between size and isotope signature for each species, and therefore account for differences in size ranges sampled across sites and years.

The number of unique sites varied from four (*A*. *baremose*) to eight (*H*. *forskalli* and *L*. *horie*) per species. The variability in any one isotopic signature (δ^13^C or δ^15^N) described by site, year and their interaction terms ranged from 2.3% (*T*. *zilli* δ^15^N) to 66.6% (*L*. *horie* δ^15^N) (Fig 5). There was no significant relationship between the variability described in δ^13^C and the variability described in δ^15^N for each species. Similarly, there was no significant relationship between the number of unique sites and the isotopic niche size of each species (S2 Fig; r^2^ = 0.1528, p = 0.3859).

### Breeding Vulnerability Index

**Fig 6:**
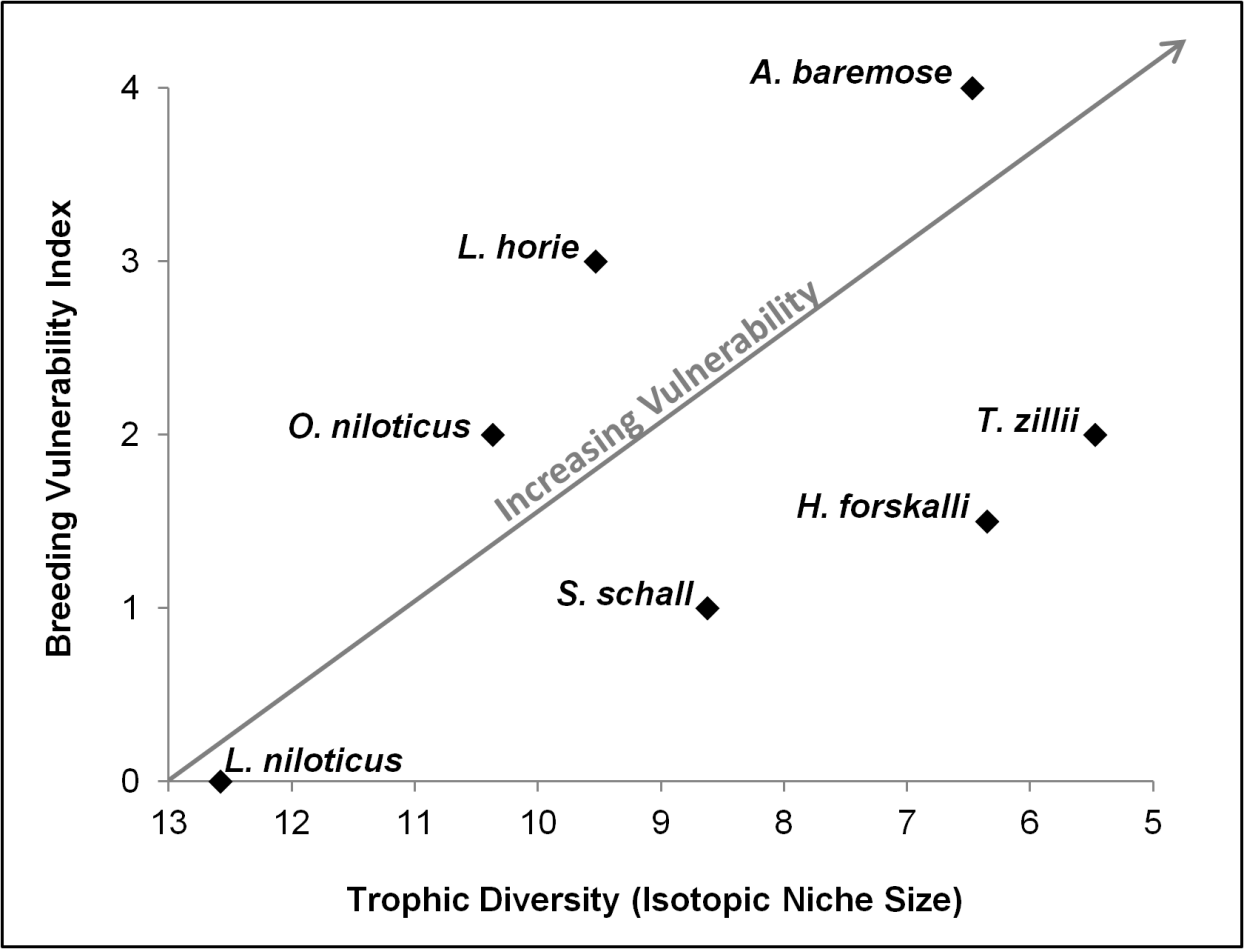
Breeding Vulnerability Index versus Trophic Diversity for the seven species studied. The Breeding Vulnerability index was calculated based on flood pulse dependence and breeding habitat requirements. Trophic Diversity (axis in reverse order) is represented by isotopic niche size, calculated using the δ^13^C and δ^15^N signatures for each species and standard ellipses in R. The grey line represents the direction of increasing vulnerability and is not a trendline.

In Lake Turkana, *A*. *baremose* and *L*. *horie* breed exclusively during the flood pulse (Flood Pulse Factor = 2), whereas *H*. *forskalli* and *O*.*niloticus* breed year-round but show marked breeding peaks during the flood pulse (Flood Pulse Factor = 1) [14]. *Lates niloticus*, *S*. *schall and T*. *zilli* show consistent breeding year-round or weak breeding peaks during the flood pulse (Flood Pulse Factor = 0). The species studied showed a wide range of breeding habitat preferences. *L*. *niloticus* breeds in the lake’s pelagic habitats (Habitat Factor = 0). Of the lake’s littoral breeders, *O*. *niloticus* prefers sandy breeding sites, which are concentrated on the lake’s western shores (Habitat Factor = 1), and *T*. *zilli* prefers rocky or macrophyte-dominated breeding sites, which are concentrated on the lake’s eastern shores (Habitat Factor = 2). Though *L*. *horie* and *S*. *schall* breed in all inflowing river mouths (Habitat Factor = 1), *A*. *baremose* breeds only in the Omo River and its Delta (Habitat Factor = 2) [14]. *H*. *forskalli* is thought to have two sub-populations, one that breeds in the lake’s pelagic habitats and one that breeds in inflowing river mouths (Habitat Factor = 0.5) [14]. The overall breeding vulnerability index ranged from 0 (*L*. *niloticus*) to 4 (*A*. *baremose*). In general, species with high trophic diversity also showed low breeding vulnerability while those with low trophic diversity also showed high breeding vulnerability (Fig 6).

## Discussion

Lake Turkana is a system expected to undergo substantial changes in the next decade due to the impact of upstream development projects and global climate change on the volume and patterns of inflow from the Omo River. The methodology we employed allows for a relatively inexpensive and quick way of developing an initial understanding of dietary position and niche breadth for key fishes of Lake Turkana. Our initial expectations regarding the dietary niche breadth of these species were structured around their trophic ecomorphology. Based on these expectations, we postulated that the dietary niches of the species studied would be (1) similar in size but (2) non-overlapping. The first hypothesis can be rejected based on the SIBER results, as isotopic niche size varied by a factor of two among the species studied. Isotopic niches for *L*. *niloticus* and *O*. *niloticus* were the largest, significantly larger than the smallest exhibited by *A*. *baremose*, *H*. *forskalli* and *T*. *zillii*. *L*. *horie* and *S*. *schall* had mid-range isotopic niches, with lower certainty regarding relative niche size based on Bayesian interference. The degree of trophic diversity as measured by SIBER was confirmed by Layman’s metrics (Hull_b_ and measures of dispersion). Although previous work has hypothesized that high trophic level species may be more likely to exhibit intraspecific variation [84,107], we did not find this to be the case among the seven species studied.

The apparent discrepancy between ecomorphology and feeding behavior, and in particular the presence of morphological specialists behaving as generalists, is not a phenomenon unique to this system. This mismatch- especially common in teleost fishes- is referred to as “Liem’s Paradox” and was first noted in east African lake cichlid fish populations [108–113]. In “Liem’s Paradox” structural feeding specializations invoke minimal functional limitations, permitting morphologically specialized taxa to feed on whatever is most advantageous when food resources are abundant and as specialists when the most desirable food resources are scarce [112,114]. In tropical freshwater systems it is the flood cycle, rather than seasonality in any strict sense (as at high latitude), that controls food resource availability. Our sampling schedule has likely captured the magnitude of each species’ intraspecific diet variation, as we sampled during periods of low lake level (i.e. resource scarcity) and high lake level (i.e. resource abundance) at both seasonal and inter-annual scales.

The SIBER results supported our second hypothesis regarding the spacing of our key species’ dietary niches in isotopic space. Except for *S*. *schall*, which overlapped with all other species, the species in this study showed little trophic overlap and are quite distinct in trophic function in Lake Turkana. High overlap between *S*. *schall* and the isotopic signatures of the other species studied indicate an omnivorous diet and a catholic behavioral repertoire for this species. In isotopic space, there was distinct separation between pelagic and littoral species. *A*. *baremose* and *H*. *forskalli* were end members representing the pelagic food web, showing the highest δ^15^N values (possibly due to a longer food web in the lake’s open waters), but the lowest δ^13^C values, indicative of pelagic food sources [104]. In contrast, *T*. *zillii* and *O*. *niloticus* had low δ^15^N values and the highest δ^13^C values, placing them as secondary consumers in the lake’s littoral food web [104]. *T*. *zillii* had the highest δ^13^C values, consistent with a diet predominantly consisting of macrophytes. The benthivores *S*. *schall* and *L*. *horie*, while not overlapping much, fell somewhere in the middle of the isotopic space. *L*. *niloticus*’ niche also fell between the pelagic and littoral end members, suggesting a mix of prey from both habitats, but also surprisingly low in trophic level given its piscivorous reputation. Relative positions of these species in isotopic space were largely in agreement with stable isotope work conducted on other African Lake ecosystems [39–42,115–116].

We recognize that variability in the isotopic baseline propagates up food webs and must be considered in understanding variability at higher trophic levels [46,51,83]. Although there are no appropriate primary consumer baseline organisms in Lake Turkana [92], the results from our primary producer baseline organisms suggest no significant difference in signatures across sites. Furthermore, factors relating to baseline differences (i.e. site and year) did not describe a consistent amount of variability among the species studied or between the isotopes studied for each species. Bearhop et al. [46] describe four factors that have a large influence on intraspecific variability in isotope signature: the range and evenness of prey items consumed, the trophic level of these prey items, and the geographic range in which a species forages. If spatial differences in baseline signature are a key component of the variability in isotopic signature of the species studied, species that were sampled at a greater number of unique sites would have larger isotopic niches on average [46]. We did not, however, find a significant relationship between the number of unique sites sampled and the isotopic niche size of the species studied, suggesting that differences in the first three factors (range, evenness and trophic level of prey items consumed) have a stronger influence on intraspecific isotopic variability in our system.

Our conclusions regarding the relative diet variability of the species studied are also corroborated by previous research on Lake Turkana and other systems. *A*. *baremose* and *H*. *forskalli* were among the more specialized feeders in recent research on Lake Albert, the African Lake with the closest fish assemblage to Lake Turkana [42]. Similarly, *T*. *zillii* is one of the most specialized feeders among the tilapias [42], and in Lake Turkana it has a strong preference for rocky littoral habitats with macrophytic vegetation [7,14,26]. Of the less specialized species, *L*. *horie*’s diet has not been extensively studied but past research on Lake Turkana suggests that it is relatively omnivorous [14], feeding on benthic items including detritus and ostracod shells, a diet consistent with this species’ position in isotopic space in this study. *S*. *schall* shows high morphological variability in Lake Turkana (i.e. color and size of spots), consumed a variety of prey items in a recent gut content study (KMFRI 2008) and has been noted as an omnivore in other systems [32–35]. It is also the only *Synodontis* species in Lake Turkana proper (*Synodontis frontosa* is confined Omo River and its delta), so it does not have competition from other members of the genus that are better suited for open water feeding (Hopson et al. 1982). *L*. *niloticus* has a wide range of isotope values in other African lakes and has been shown to prey-switch in some systems, suggesting that it is an opportunistic feeder [42,92,107,116–117]. Similarly, *O*. *niloticus* is known to be a particularly plastic species in ecosystems worldwide, capable of withstanding high environmental fluctuations and extreme breadth in both its fundamental and realized trophic niche [7].

To better predict how these species will respond to changes in the ecosystem, we considered our intraspecific trophic diversity results in the context of breeding vulnerability. Interestingly, our most vulnerable species based on diet (i.e. small isotopic niche, low intraspecific trophic diversity) also tended to have high breeding vulnerabilities (Fig 6). We predict that, in suffering two strikes against them, *A*. *baremose*, *H*. *forskalli*, *L*. *horie*, and *T*. *zillii* will be the losers in a greatly altered Lake Turkana. These predictions are largely in agreement with historical changes in the lake, as *A*. *baremose* and *H*. *forskalli* both showed sharp population declines when lake levels dropped between the 1970’s and 1980’s. *L*. *horie* did not show similar declines, but a reduced catch of juveniles did suggest recruitment failure [24]. *L*. *niloticus* and *O*. *niloticus* are expected to be the winners (i.e. fare better than the other species studied in the face of change) in the first phase of changes to Lake Turkana. These species both have low breeding vulnerability and high intraspecific trophic diversity (i.e. extralimital feeding adaptations, large isotopic niche), and have been highly successful invasive species in other systems, suggesting a general hardiness and ecological flexibility. We also group *S*. *schall* with the predicted winners in this ecosystem, based on its low breeding vulnerability and highly overlapping isotopic niche, consistent with an omnivorous diet. Although this species breeds primarily in inflowing rivers, it is not dependent solely on the Omo River and there is some evidence that it may also breed in shallow, sandy habitats within the lake proper [14]. There was no evidence for population declines in *L*. *niloticus* or *S*. *schall* during the lake level declines of the 1970’s-1980’s [24].

In some ways, it is promising that *O*. *niloticus* and *L*. *niloticus* are among the less vulnerable species in Lake Turkana. Together, their multiple size classes can fill out a complete, if depauperate, fish food web. They are also highly desirable fisheries resources, and are the most valuable and heavily exploited species in the growing Lake Turkana fishery. In contrast, *L*. *horie* is the third largest fishery on Lake Turkana but based upon our results will be among the most sensitive to imminent changes in the ecosystem, with implications for food security and quality. *T*. *zillii* should be treated as separate from *O*. *niloticus* in the fishery, as our results suggest that these two species play different ecological roles in Lake Turkana. *T*. *zillii* will also be far less resilient to perturbation than *O*. *niloticus* and should be managed accordingly. Fishermen should be able to separate these species quickly in the field, given highly visible differences in morphology and coloration.

Past researchers have advocated for growth in the lake’s offshore fishery, which would focus on *H*. *forskalli* and *A*. *baremose* [14]. It is unlikely that these two species will be able to withstand the combined effects of lake level decline and increased fishing pressure, given historic declines in their populations that occurred without the added impacts of fishing [24]. We therefore cannot recommend that a fishery for these species be developed on Lake Turkana. *S*. *schall* was among the more resilient species in this study and is one of the most abundant fish species in the Lake Turkana ecosystem [14,36]. Dietary studies suggest little predation pressure on *S*. *schall* due to its formidable morphological defense of interlocking pectoral spines, so this species may as a food web “dead end” [1,14,37]. As earlier suggested by [24], we believe there may be potential for sustainably increasing fishing pressure on this species. However, an expanded fishery for *S*. *schall* would require careful monitoring and development and enforcement of measures to limit the bycatch of more sensitive species.

In general, there is a lack of enforcement of fisheries regulations on Lake Turkana due to insufficient staff numbers and funding among local research and management agencies. This reality will make heeding the recommendations above a difficult task. Overexploitation coupled with environmental sensitivity has led to the decline of some species in the past, including the collapse of the *C*. *citharus* and *D*. *niloticus* fishery between the 1970’s and 1980’s [24]. Even among the species that we consider to be least vulnerable there are concerns of overexploitation and subsequent declines (Ojwang, *pers*. *obs*.). These species will only be true winners in the ecosystem if they are managed sustainably. The fishery for *O*. *niloticus* in particular is unrelenting, with fishermen targeting this species by day and night in shallow areas using seine nets. Lake Turkana’s catches are relatively minor compared to Lake Victoria’s but play an important role in local food security. Furthermore, there is the potential to increase the lake’s fishery if done so sustainably, particularly if the fishery could include less valuable but highly productive species like *S*. *schall*. To ensure that Lake Turkana’s fishery does not collapse in this period of multiple stressors, effort should be made to increase the resources available to local organizations monitoring the system.

## Conclusions

Local dam and irrigation development and global climate change will alter the Omo River’s flow patterns over the coming decade, with a possibility of terminating freshwater inflow to Lake Turkana, dropping lake level, and dampening intra-annual fluctuations. Our results suggest that the winners in Lake Turkana will be *L*. *niloticus*, *O*. *niloticus*, and *S*. *schall*, which together make up 40% of recent fisheries catch. *S*. *schall* has shown low predation mortality in previous diet studies on the system [1,14] and the fishery for this species could be expanded sustainably if measures are taken to avoid bycatch of other species. Unlike *O*. *niloticus*, *T*. *zillii* is a limited-specialist feeder (*O*. *niloticus* is a generalist with a non-limiting specialization for microphagy; [118]) and will be particularly vulnerable to breeding habitat changes, so these species should not be grouped by the fishery simply because they are both tilapiines. After *L*. *niloticus* and *O*. *niloticus*, *L*. *horie* is the lake’s most important fishery resource and is also one of the least resilient species, or losers, based on our study. *H*. *forskalli* is likely to feed primarily on *A*. *baremose* and related species and both *H*. *forskalli* and *A*. *baremose* will be losers in the altered Lake Turkana due to their dietary constraints and high breeding vulnerability. In general, fishery management in the region must be improved to account for potential population declines among vulnerable species and to sustainably manage resilient species so that they stay winners. Although additional research is needed to fully understand this understudied and climate sensitive ecosystem, our study serves as a robust initial prediction of how Lake Turkana’s fish community will be altered by upstream development, contributes to a still wanting store of baseline data for the ecosystem, and provides a novel use of data on intraspecific diet variation to predict sensitivity to anthropogenic impacts.

## Supporting Information

S1 Fig Boxplot of attached primary producer isotope signatures at three of the study’s littoral sites. Hippograss, *Echinochloa stagnina*, was the only species found at multiple sites. There was no significant difference in the signature of *Echinochloa stagnina* between Sibiloi and Ferguson’s Gulf.
(TIF)

S2 Fig The relationship between the number of unique sites and isotopic niche size for each species. The number of unique sites was defined as any unique combinations of site and year at which >5 individuals of a species were sampled. There was no significant relationship beween number of unique sites and isotopic niche size across the species studied (r^2^ = 0.1528, p = 0.3859).
(TIF)
