# Supplementary figures and images for: Predicting Species’ Vulnerability in a Massively Perturbed System: The Fishes of Lake Turkana, Kenya
Source: PLoS One. 2015 May 19;10(5):e0127027. doi: 10.1371/journal.pone.0127027 (PMC4437984; doi:10.1371/journal.pone.0127027)

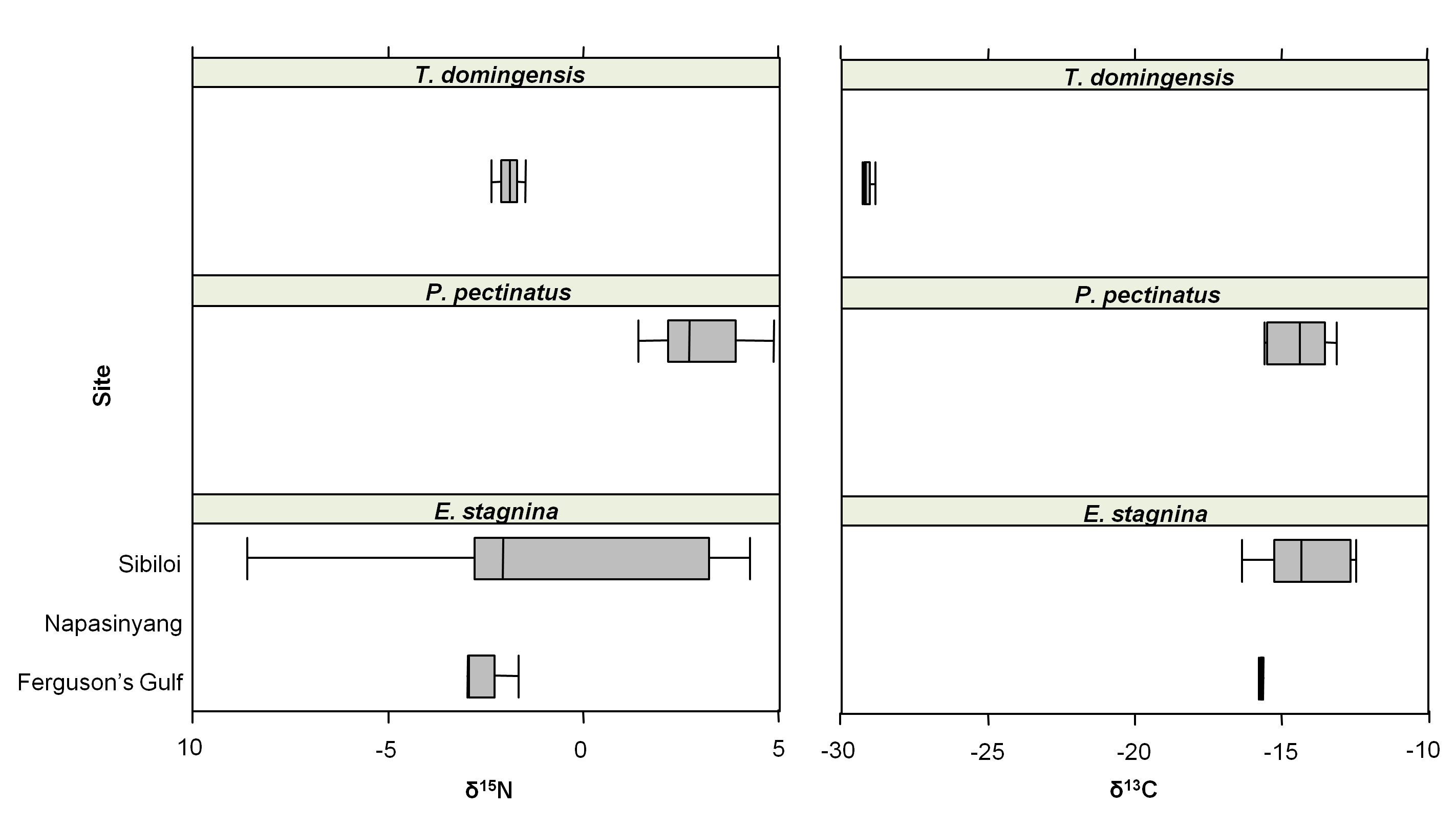

Supplement: S1 Fig — Hippograss, Echinochloa stagnina, was the only species found at multiple sites. There was no significant difference in the signature of Echinochloa stagnina between Sibiloi and Ferguson’s Gulf. (TIF) [file pone.0127027.s001.tif]

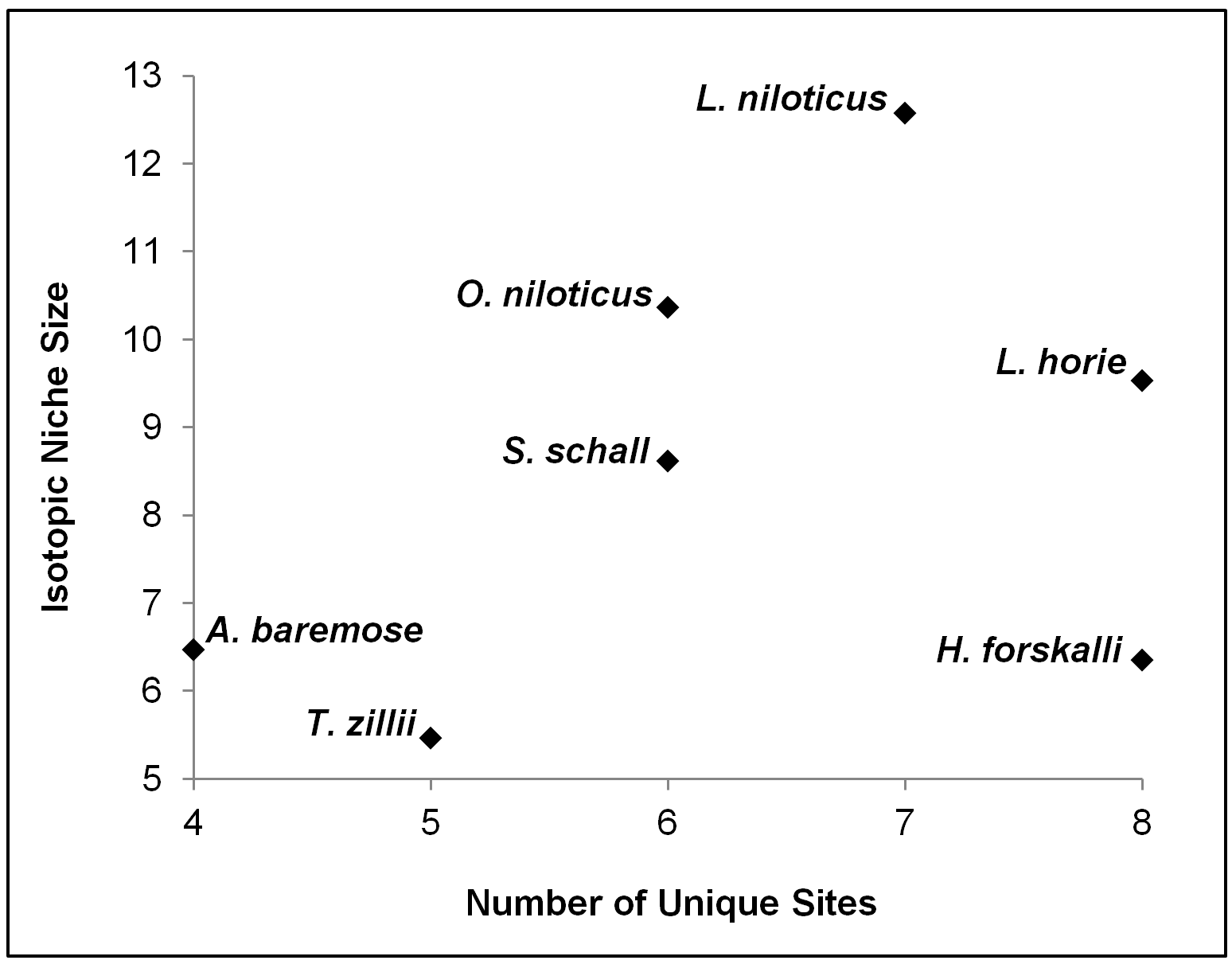

Supplement: S2 Fig — The number of unique sites was defined as any unique combinations of site and year at which >5 individuals of a species were sampled. There was no significant relationship beween number of unique sites and isotopic niche size across the species studied (r2 = 0.1528, p = 0.3859). (TIF) [file pone.0127027.s002.tif]
